# Robotic Care Outcomes Project (ROBOCOP) for elective cholecystectomy

**DOI:** 10.1007/s00464-025-12109-1

**Published:** 2025-08-26

**Authors:** Marc Abou Assali, Yanli Li, Hannah Bossie, Christopher Neighorn, Esther Wu, Kaushik Mukherjee

**Affiliations:** 1 Department of Surgery, Loma Linda University Health, Loma Linda, CA USA; 2 Intuitive Surgical Inc, Sunnyvale, CA USA; 3 Division of General and Gastrointestinal Surgery, Loma Linda University Health, Loma Linda, CA USA; 4 Division of Acute Care Surgery, Loma Linda University Health, 11175 Coleman Pavilion, CP 21111, Loma Linda, CA 92350 USA

**Keywords:** Robotic cholecystectomy, Laparoscopic cholecystectomy, Conversion to open surgery, Bile duct injury, Elective surgery

## Abstract

**Background:**

Robotic cholecystectomy (RCHOLE) is being used more frequently for elective patients. We aimed to compare clinical outcomes, specifically conversion to open/subtotal cholecystectomy, for RCHOLE and laparoscopic cholecystectomy (LCHOLE).

**Methods:**

Our study received a Non-Human Subjects Research Determination. We studied elective laparoscopic (LCHOLE) and robotic (RCHOLE) cases from 2020 to 2022 using de-identified extraction of electronic US hospital health record data from the Intuitive Custom Hospital Analytics database. LCHOLE and RCHOLE cases, conversion to open/subtotal cholecystectomy, and complications were identified using ICD10 and/or CPT codes. Patients with missing operative times and demographics were excluded (n = 11,276). We used Multivariate Logistic Regression with Inverse Probability Treatment Weighting(MLR/IPTW) to balance covariates. R 4.1.1 was used for analysis.

**Results:**

LCHOLE(n = 93,122) and RCHOLE(n = 23,581) had similar mean age(50 years) and gender(70% female); RCHOLE patients were more frequently obese(BMI ≥ 30 kg/m2, 38.0% vs. 33.4%, *p* < 0.001). Operative time was longer in RCHOLE(107 ± 53 vs. 93 ± 42 min, *p* < 0.001). After MLR/IPTW, RCHOLE had decreased odds of conversion to open cholecystectomy (OR 0.51 [95%CI 0.42, 0.61, *p* < 0.001), but similar odds of subtotal cholecystectomy. Readmission (OR 0.89 [0.81, 0.97, *p* = 0.008]) hospital acquired conditions (OR 0.71 [0.60, 0.83, *p* < 0.001]), and bile duct injury (OR 0.00, *p* < 0.001) were less likely with RCHOLE. Odds of surgical site infection and hospital mortality were similar in both groups.

**Conclusions:**

In the elective setting, robotic cholecystectomy has reduced odds of conversion to open, readmission, and hospital acquired conditions including bile duct injury when compared to laparoscopic cholecystectomy.

**Supplementary Information:**

The online version contains supplementary material available at 10.1007/s00464-025-12109-1.

The gold standard for treatment of gallbladder disease is laparoscopic cholecystectomy, (LCHOLE) which is one of the most common surgeries performed worldwide and is associated with reduced perioperative complications and length of stay when compared to open cholecystectomy [1–4]. However, conversion to open cholecystectomy remains steadily in the 4–6% range, with converted cases experiencing longer length of stay, more perioperative complications, and higher costs than cases that are completed laparoscopically [5–9].

Robotic cholecystectomy has some potential technological advantages that may provide an enhanced ability to complete complex cases while remaining minimally invasive, including increased dexterity, improved three-dimensional visualization, reduction of tremor, scaling of surgeon motion, and complete integration of indocyanine green fluorescent angiography [10–12]. We have previously demonstrated that, in the setting of acute gallbladder pathology, robotic cholecystectomy significantly reduced conversion rate to open cholecystectomy but increased the odds of subtotal cholecystectomy [13]. However, this is still a controversial topic, as the recent manuscript by Kalata and coauthors raised concerns about the rate of bile duct injuries sustained during robotic cholecystectomy procedures in a large insurance database. Of note, the Kalata manuscript did not address conversion to open surgery, and the rate of bile duct injury in open cholecystectomies was 4% in this study [14]. Although the study cited learning curve as a reason for decreasing bile duct injuries in the robotic surgery arm with time, the same finding was noted in the laparoscopic and open cholecystectomies [14].

Given the inconsistent results seen in many comparative studies, this study aims to investigate clinical outcome differences between elective laparoscopic and robotic cholecystectomies, specifically identifying rates of conversion to open/subtotal cholecystectomy and associated complications, using a national database encompassing multiple institutions. We hypothesize that the robotic cholecystectomy group will have improved clinical outcomes and rates of conversion to an open or subtotal technique when compared to the laparoscopic group.

## Methods

### IRB approval

This project was reviewed by the institutional review board at the authors’ institution and received a designation of non-human subjects research, and as such was considered to not require further review. We followed the EQUATOR guidelines as applicable to case–control studies (STROBE checklist), which is attached as supplemental digital content. (SDC 1).

### Study population

A retrospective study was conducted to evaluate perioperative outcomes of minimally invasive cholecystectomies using the Intuitive Custom Hospital Analytics (CHA) database. The CHA database extracted de-identified, HIPAA compliant patient data from hospital electronic health records (EHR) in the US under non-disclosure agreement. Facilities self-select to opt into this database to understand national benchmarks, performance improvement and support quality initiatives. Individual hospitals conducted data validation and integrity checks prior to submitting their data to the CHA database. Any protected health information was de-identified by individual hospitals before data transmission.

Adult patients who received elective minimally invasive cholecystectomies from 2020 to 2022 were included in the study. Minimally invasive cholecystectomy was defined using the International Classification of Disease (ICD Tenth Revision, Clinical Modification (ICD-10-CM) procedure codes and Current Procedural Terminology (CPT) codes (SDC 2). Laparoscopic cholecystectomies (LCHOLE) and robotic cholecystectomies (RCHOLE) were differentiated by hospital provided identifiers. Patients with missing operative time or some basic demographic and clinical information (e.g., gender, emergent/elective cases, setting of care, region) were excluded.

### Covariates and outcomes

Patient-level demographic and clinical characteristics included age, sex, indication for surgery, body mass index (BMI), presence of diabetes, payor type and year of surgery. Hospital region and teaching status were also included as covariates. Clinical outcomes being evaluated were conversion to open surgery or subtotal cholecystectomy, blood transfusions, in-hospital mortality, perioperative complications including surgical site infection, bile duct injury or leak, pancreatic leak/fistula, other postprocedural complications, and hospital acquired conditions per the Center for Medicare and Medicaid Services (https://www.cms.gov/medicare/payment/fee-for-service-providers/hospital-aquired-conditions-hac/hospital-aquired-conditions). ICD-10-CM diagnosis codes were used to define the outcomes of interest (SDC 2). Operative room (OR) time and hospital length of stay data were extracted from the hospital EHR. Annual surgeon volume of cholecystectomy procedures was obtained and divided into the lowest tertile (low volume) versus mid and high tertiles (non-low volume) to attempt to analyze the effect of surgeon learning curve. Cutoff for the lowest tertile was 35 cases per year. Activity based costing was used to perform a cost analysis as previously described [13].

### Statistical analysis

Inverse probability treatment weighting (IPTW) using stabilized weights was performed to balance distributions of covariates in LCHOLE and RCHOLE patients. Inverse probability of treatment weights were calculated as the inverse of patients’ estimated probability of receiving different surgical modality using a logistic regression model including all the aforementioned baseline factors. Distributions of baseline characteristics in both the raw data and IPTW data were presented. Adequacy of IPTW to balance covariates was assessed by using absolute value of standardized mean difference (SMD) less than 0.1 [1, 2].

Logistic regressions were performed in the raw data and IPTW data, to compare the clinical outcomes of LCHOLE and RCHOLE patients, respectively. Odds ratio (OR) and 95% confidence intervals (95% CIs) were calculated. Additionally, a multivariate logistic regression analysis was performed to identify risk factors for conversion to open surgery. All analyses were performed using R version 4.2.2. Two-tailed p-values less than 0.05 were considered statistically significant.

## Results

**Fig. 1:**
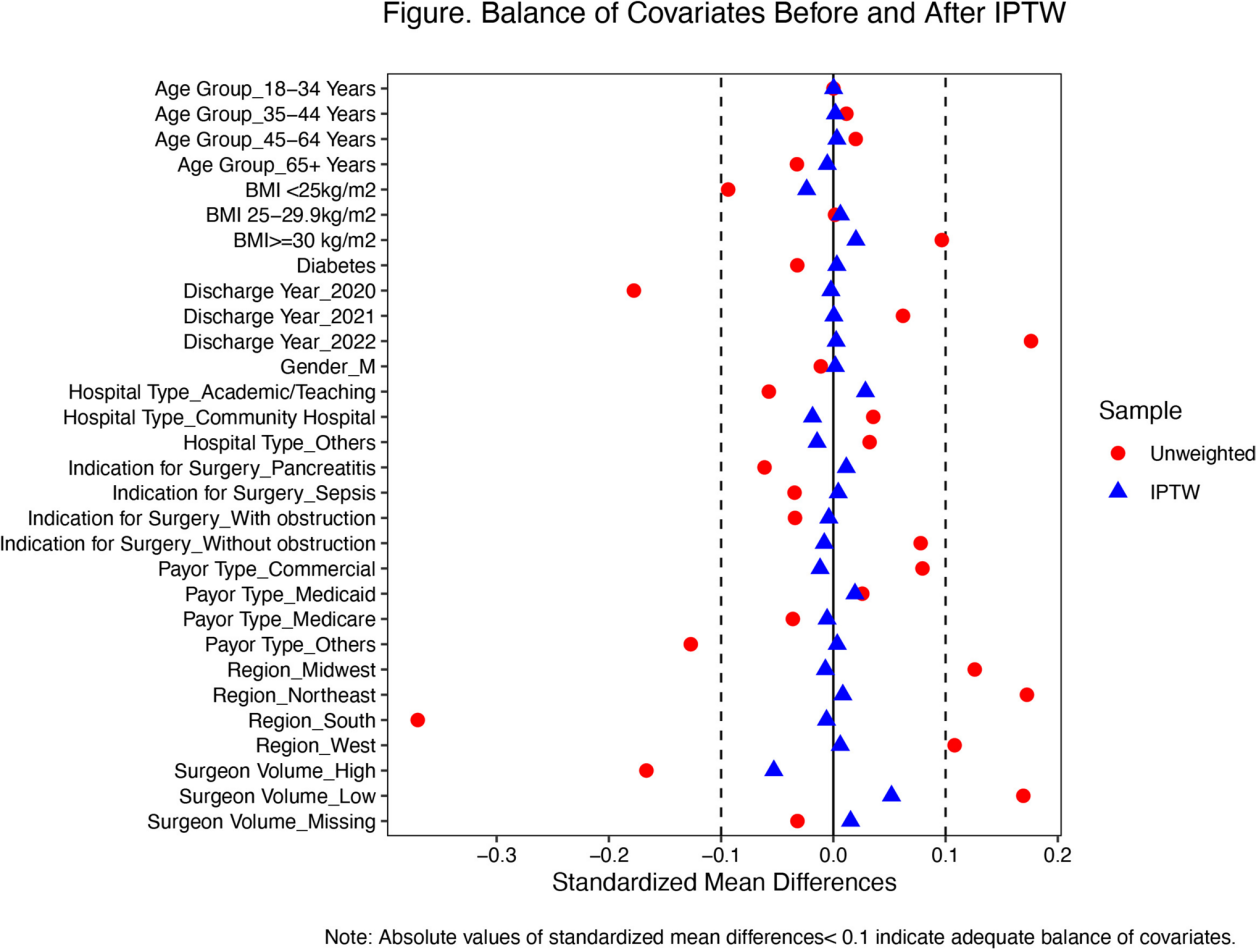
The Inverse Probability Treatment Weighting (IPTW) algorithm is used to balance the covariates between the laparoscopic and robotic cholecystectomy groups. The unweighted standardized mean differences are indicated in red circles and the post-IPTW standardized mean differences are in blue triangles

A total of 128,047 patients who underwent elective LCHOLE or RCHOLE from 2020 to 2022 were identified and 116,771 patients remained after excluding patients with missing values. There were 93,087 LCHOLE and 23,684 RCHOLE included in the study, which became 93,122 LCHOLE and 23,581 RCHOLE after weighed analysis with IPTW. Gender, age group, indication for surgery, BMI, diabetes diagnosis, discharge year, hospital type, payor type, hospital region were accounted for with adequate balance according to standardized mean difference values obtained (Table 1, Fig. 1).

**Table 1:** Basic information of elective cholecystectomy before and after IPTW

	Unweighted			IPTW		
Characteristic	Lap, N = 93,087^1^	Robotic, N = 23,684^1^	SMD^2^	Lap, N = 93,122^1^	Robotic, N = 23,581^1^	SMD^2^
Gender						
F	64,743 (69.55%)	16,594 (70.06%)		64,859 (69.65%)	16,405 (69.57%)	
M	28,344 (30.45%)	7090 (29.94%)	− 0.0112	28,262 (30.35%)	7176 (30.43%)	0.0018
Age group						
18–34 years	21,384 (22.97%)	5442 (22.98%)	0.0001	21,395 (22.98%)	5420 (22.99%)	0.0003
35–44 years	15,711 (16.88%)	4101 (17.32%)	0.0116	15,805 (16.97%)	4019 (17.04%)	0.0018
45–64 years	32,525 (34.94%)	8501 (35.89%)	0.0199	32,721 (35.14%)	8321 (35.29%)	0.0031
65 + years	23,467 (25.21%)	5640 (23.81%)	−0.0325	23,200 (24.91%)	5820 (24.68%)	− 0.0054
Indication for surgery						
Without obstruction	89,182 (95.80%)	23,028 (97.23%)	0.0778	89,482 (96.09%)	22,624 (95.94%)	− 0.0081
Pancreatitis	1743 (1.87%)	267 (1.13%)	− 0.0613	1605 (1.72%)	440 (1.86%)	0.0116
Sepsis	1174 (1.26%)	214 (0.90%)	− 0.0346	1107 (1.19%)	291 (1.23%)	0.0044
With obstruction	988 (1.06%)	175 (0.74%)	− 0.0341	927 (1.00%)	226 (0.96%)	−0.0040
Overweight or obesity						
Not overweight or obesity	48,117 (51.69%)	11,134 (47.01%)	− 0.0937	47,194 (50.68%)	11,671 (49.49%)	− 0.0238
Obesity	31,095 (33.40%)	9007 (38.03%)	0.0966	32,028 (34.39%)	8337 (35.36%)	0.0201
Overweight	13,875 (14.91%)	3543 (14.96%)	0.0015	13,900 (14.93%)	3572 (15.15%)	0.0063
Diabetes	11,491 (12.34%)	2,678 (11.31%)	− 0.0321	11,304 (12.14%)	2886 (12.24%)	0.0031
Discharge year						
2020	45,916 (49.33%)	9597 (40.52%)	− 0.1777	44,261 (47.53%)	11,183 (47.42%)	− 0.0021
2021	38,518 (41.38%)	10,527 (44.45%)	0.0620	39,108 (42.00%)	9909 (42.02%)	0.0005
2022	8653 (9.30%)	3560 (15.03%)	0.1762	9,753 (10.47%)	2,489 (10.56%)	0.0025
Hospital type						
Academic/Teaching	9,284 (9.97%)	1,970 (8.32%)	− 0.0575	8,994 (9.66%)	2,472 (10.48%)	0.0286
Community hospital	81,646 (87.71%)	21,044 (88.85%)	0.0356	81,888 (87.94%)	20,596 (87.34%)	− 0.0185
Others	2157 (2.32%)	670 (2.83%)	0.0323	2240 (2.41%)	513 (2.18%)	− 0.0145
Payor type						
Commercial	43,264 (46.48%)	11,947 (50.44%)	0.0794	44,009 (47.26%)	11,005 (46.67%)	-0.0118
Medicaid	16,293 (17.50%)	4,380 (18.49%)	0.0258	16,524 (17.74%)	4,359 (18.48%)	0.0193
Medicare	24,801 (26.64%)	5,936 (25.06%)	− 0.0361	24,496 (26.31%)	6,146 (26.06%)	− 0.0056
Others	8,729 (9.38%)	1,421 (6.00%)	-0.1270	8,092 (8.69%)	2,071 (8.78%)	0.0035
Region						
Northeast	12,639 (13.58%)	4737 (20.00%)	0.1725	13,884 (14.91%)	3590 (15.22%)	0.0084
Midwest	22,290 (23.95%)	6989 (29.51%)	0.1260	23,335 (25.06%)	5835 (24.75%)	− 0.0071
South	35,982 (38.65%)	5,192 (21.92%)	− 0.3703	32,809 (35.23%)	8243 (34.96%)	− 0.0061
West	22,176 (23.82%)	6766 (28.57%)	0.1081	23,094 (24.80%)	5912 (25.07%)	0.0062
Surgeon volume						
Low volume (≤ 35/yr)	31,538 (33.88%)	9963 (42.07%)	0.1693	33,272 (35.73%)	9016 (38.24%)	0.0518
High volume (> 35/yr)	61,351 (65.91%)	13,700 (57.84%)	− 0.1666	59,674 (64.08%)	14,506 (61.52%)	− 0.0530
Missing	198 (0.21%)	21 (0.09%)	− 0.0320	175 (0.19%)	58 (0.25%)	0.0154

^1^ n (%)

^2^ *SMD* standardized mean difference. Absolute values of standardized mean differences < 0.1 indicate adequate balance

Unadjusted data is presented in Table 2. Conversion to open surgery rate was lower in RCHOLE when compared to LCHOLE (0.58% vs. 1.22%, *p* < 0.001). After a conversion to open surgery occurs, a reduced likelihood of completing a subtotal cholecystectomy was observed for RCHOLE patients (OR = 0.13 [0.03, 0.52]). Overall rates of subtotal cholecystectomy were similar between groups, but rates of conversion to either open or subtotal cholecystectomy were lower for RCHOLE (0.77% vs. 1.40%, *p* < 0.001). Readmission was lower in RCHOLE (2.95% vs. 3.34%, *p* = 0.003), as were hospital acquired conditions (0.85% vs. 1.37%, *p* < 0.001), bile duct injuries ( 0.00% vs. 0.02%, *p* = 0.009)and length of stay (1.68 ± 2.18 vs. 1.89 ± 2.57 days, *p* < 0.001). Operative time was longer in RCHOLE (107 ± 53 vs. 93 ± 42 min, *p* < 0.001). Cost was lower in the RCHOLE group ($8007 ± 10,378 vs. $8572 ± 13,972, *p* < 0.001).

**Table 2:** Crude outcomes of elective cholecystectomy patients by modality

	Lap, N = 93,087^1^	Robotic, N = 23,684^1^	p-value
Subtotal/Chole			0.30
Cholecystectomy	92,872 (99.77%)	23,638 (99.81%)	
Subtotals	215 (0.23%)	46 (0.19%)	
Conversion to open			** < 0.001**
Not converted to open	91,949 (98.78%)	23,546 (99.42%)	
Converted to open	1,138 (1.22%)	138 (0.58%)	
Conversion to open or subchole			** < 0.001**
Non-converted	91,785 (98.60%)	23,502 (99.23%)	
Converted to open	1087 (1.17%)	136 (0.57%)	
Converted to subtotal	164 (0.18%)	44 (0.19%)	
Converted to open but do subtotal	51 (0.05%)	2 (0.01%)	
Any conversion			** < 0.001**
Not converted	91,785 (98.60%)	23,502 (99.23%)	
Converted to open or subtotal	1,302 (1.40%)	182 (0.77%)	
Surgical site infection	49 (0.05%)	19 (0.08%)	0.12
Blood transfusion	318 (0.34%)	66 (0.28%)	0.13
Readmission	3109 (3.34%)	699 (2.95%)	**0.003**
Hospital acquired conditions^2^	1276 (1.37%)	201 (0.85%)	** < 0.001**
Bile duct injury	15 (0.02%)	0 (0.00%)	**0.009**
Other postprocedural complications	186 (0.20%)	35 (0.15%)	0.10
Pancreatic leak/fistula	122 (0.13%)	34 (0.14%)	0.60
Mortality in hospital	55 (0.06%)	13 (0.05%)	0.80
Length of stay			** < 0.001**
Mean (SD)	1.89 (2.57)	1.68 (2.18)	
Median (IQR)	1.00 (1.00, 2.00)	1.00 (1.00, 1.00)	
Operative time			** < 0.001**
Mean (SD)	93 (42)	107 (53)	
Median (IQR)	83 (66, 108)	95 (76, 122)	
Total ABC cost			** < 0.001**
Mean (SD)	8572 (13,972)	8007 (10,378)	
Median (IQR)	4728 (3,214, 9,739)	4751 (3,564, 8,142)	

Bolded p-values indicate statistical significance with *p* < 0.05

^1^ n (%)

^2^Hospital acquired conditions include foreign object retained after surgery, stage III and IV pressure ulcers, deep vein thrombosis (DVT)/pulmonary embolism (PE), catheter-associated urinary tract infection (UTI), vascular catheter-associated infection and iatrogenic pneumothorax with venous catheterization

**Fig. 2:**
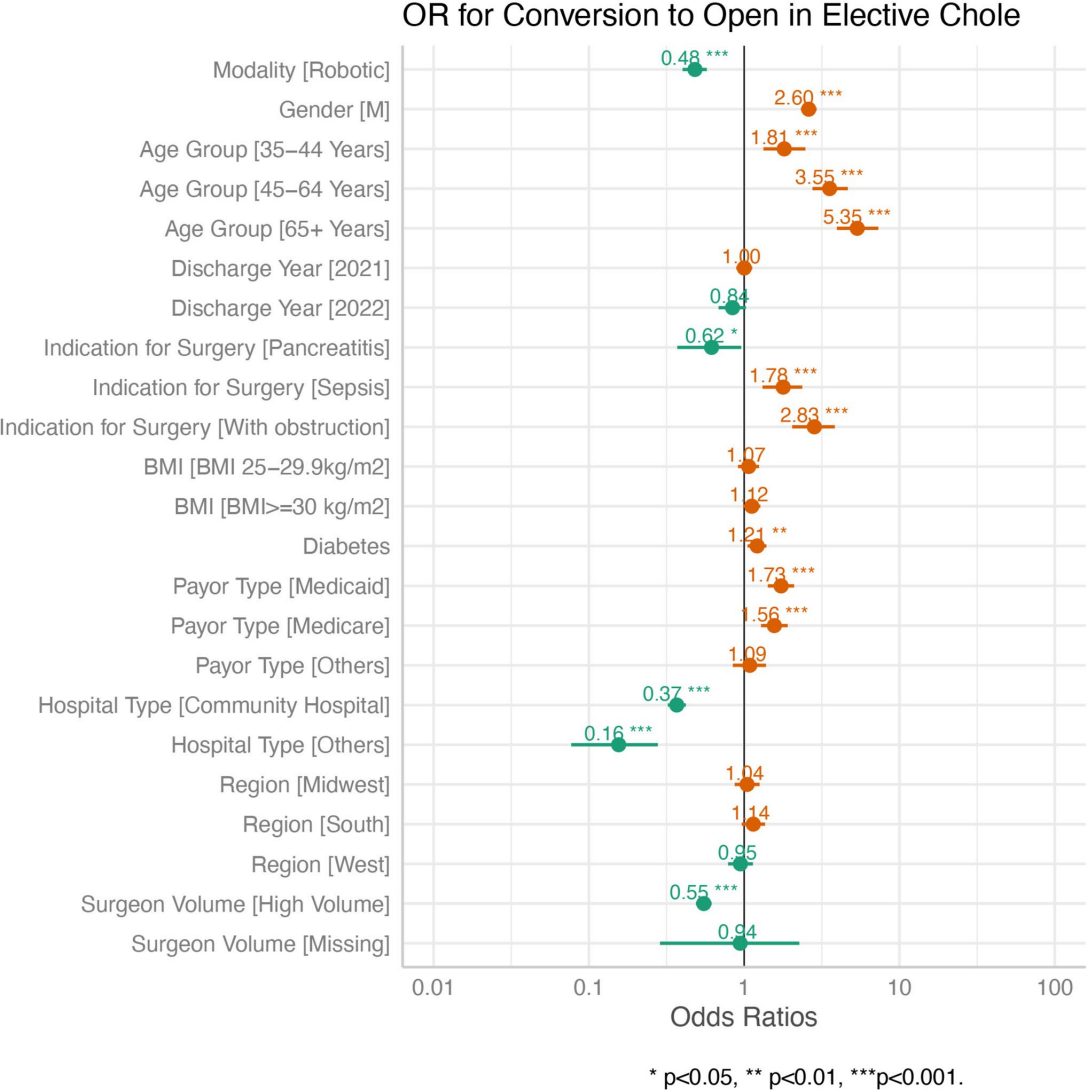
Multivariate logistic regression is used to generate odds of conversion to open surgery based on covariates. Covariates in green, with an odds ratio less than 1, are less likely to result in conversion, whereas covariates in amber, with an odds ratio greater than 1, are more likely to result in conversion. The number of asterisks after each odds ratio illustrate the level of significance

**Fig. 3:**
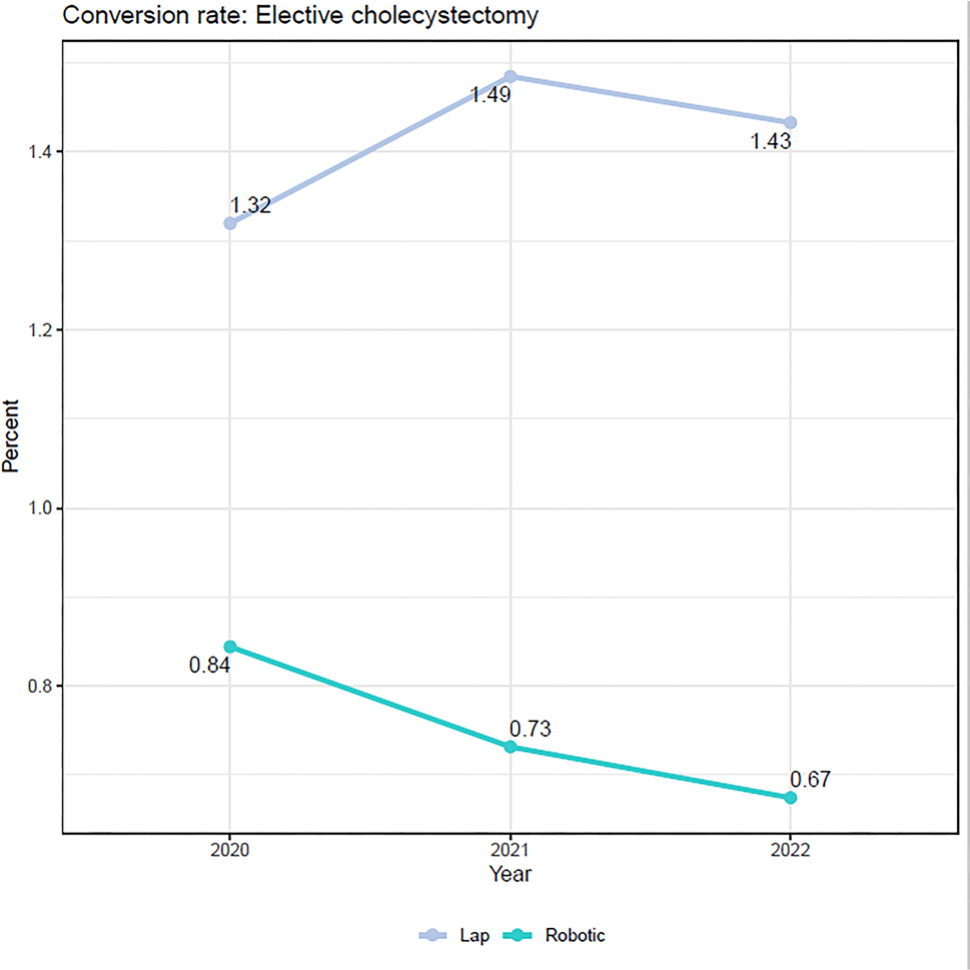
Conversion rates for laparoscopic (top) and robotic (bottom) cholecystectomy are graphed annually. The overall trend in robotic cholecystectomy coversion rates illustrates a decrease, while laparoscopic conversion rates remain relatively constant

Data after adjustment with IPTW is shown in Table 3. Patients with RCHOLE had a reduced rate of conversion to open surgery (OR = 0.51 [95%CI 0.42, 0.61], *p* < 0.001). Once the conversion to open surgery was accomplished, there was reduced chance of doing a subtotal cholecystectomy with RCHOLE (OR = 0.13 [0.03, 0.52]). However, overall there was no difference in the rate of subtotal cholecystectomy between groups. Additional notable findings include lower rates of readmission (OR = 0.89 [0.81, 0.97], *p* = 0.008), bile duct injuries (OR = 0.00, [0.00, 0.00], *p* < 0.001,and hospital acquired conditions (OR = 0.71 [0.60, 0.83], *p* < 0.001) with RCHOLE. RCHOLE had a slightly shorter length of stay (1.70 ± 2.25 vs. 1.89 ± 2.55 days, *p* < 0.001) and lower cost ($8108 ± $10,702 vs. $8623 ± $14,512, *p* < 0.001), but longer operative time (108 vs. 93 min, *p* < 0.001) when compared to laparoscopy The remaining measured variables showed no statistical differences (Fig. 2). In addition, conversion rates were studied by year, and the laparosocpic conversion rate remained relatively constant while the robotic conversion rate decreased (Fig. 3).

**Table 3:** Adjusted outcomes for elective cholecystectomy using IPTW

	Lap, N = 93,122^1^	Robotic, N = 23,581^1^	OR (95% CI)^2^	p-value
Subtotal/Chole				0.60
Cholecystectomy	92,903 (99.8%)	23,530 (99.8%)	–	
Subtotals	218 (0.2%)	50 (0.2%)	0.91 (0.65, 1.29)	
Conversion to open				** < 0.001**
Not converted to open	91,991 (98.8%)	23,435 (99.4%)	–	
Converted to open	1130 (1.2%)	146 (0.6%)	0.51 (0.42, 0.61)	
Conversion to open or subchole				** < 0.001**
Non-converted	91,824 (98.6%)	23,386 (99.2%)	–	
Converted to open	1079 (1.2%)	144 (0.6%)	0.52 (0.43, 0.64)	
Converted to open but do subtotal	51 (0.1%)	2 (0.0%)	0.13 (0.03, 0.52)	
Converted to subtotal	168 (0.2%)	49 (0.2%)	1.14 (0.80, 1.64)	
Any conversion				** < 0.001**
Not converted	91,824 (98.6%)	23,386 (99.2%)	–	
Converted to open or subtotal	1,298 (1.4%)	195 (0.8%)	0.59 (0.50, 0.70)	
Surgical site infection	49 (0.1%)	19 (0.1%)	1.51 (0.87, 2.62)	0.14
Blood transfusion	316 (0.3%)	77 (0.3%)	0.96 (0.72, 1.28)	0.80
Readmission	3,087 (3.3%)	696 (3.0%)	0.89 (0.81, 0.97)	**0.008**
Hospital acquired conditions^3^	1,249 (1.3%)	224 (1.0%)	0.71 (0.60, 0.83)	** < 0.001**
Bile duct injury	15 (0.0%)	0 (0.0%)	0.00 (0.00, 0.00)	** < 0.001**
Other postprocedural complications	186 (0.2%)	33 (0.1%)	0.71 (0.49, 1.03)	0.073
Pancreatic leak/fistula	120 (0.1%)	37 (0.2%)	1.20 (0.81, 1.79)	0.40
Mortality in hospital	53 (0.1%)	15 (0.1%)	1.15 (0.60, 2.19)	0.70
Length of stay				** < 0.001**
Mean (SD)	1.89 (2.55)	1.70 (2.25)		
Median (IQR)	1.00 (1.00, 2.00)	1.00 (1.00, 1.00)		
Operative time				** < 0.001**
Mean (SD)	93 (43)	108 (55)		
Median (IQR)	83 (66, 109)	95 (76, 123)		
Total ABC cost				
Mean (SD)	8623 (14,512)	8108 (10,702)		** < 0.001**
Median (IQR)	4765 (3,232, 9,814)	4700 (3,493, 8,220)		

Bolded p-values indicate statistical significance with *p* < 0.05

^1^n (%)

^2^*OR* odds ratio, *CI* confidence interval

^3^Hospital acquired conditions include foreign object retained after surgery, stage III and IV pressure ulcers, deep vein thrombosis (DVT)/pulmonary embolism (PE), catheter-associated urinary tract infection (UTI), vascular catheter-associated infection and iatrogenic pneumothorax with venous catheterization

Additionally, a multivariate logistic regression model of cholecystectomies procedures was also conducted, consistently suggests that robotic approach was associated with decreased odds of conversion (0.48 [0.40, 0.57], *p* < 0.001) compared to laparoscopic, and demonstrates the following as risk factors for conversion to open (Table 4): male sex (2.60 [2.32, 2.93], *p* < 0.001), increasing age [35–44 years (1.81 [1.33, 2.48], *p* < 0.001), 45–64 years (3.55 [2.75, 4.65], *p* < 0.001), 65 + years (5.35 [3.95, 7.31], *p* < 0.001)], sepsis (1.78 [1.31, 2.37], *p* < 0.001) or obstruction (2.83 [2.04, 3.83], *p* < 0.001) as the indication for surgery, or diagnosis of diabetes (1.21 [1.05, 1.39], *p* = 0.007), Medicare (1.56 [1.28, 1.91] *p* < 0.001) and Medicaid patients (1.73 [1.42, 2.10], *p* < 0.001) had increased odds of conversion. Compared to academic/teaching hospitals, community (0.37 [0.33, 0.42], *p* < 0.001), or other hospital types (0.16 [0.08, 0.28], *p* < 0.001) were associated with lower odds of conversion to open surgery. Surgeons performing more than 35 cases a year were less likely to have conversion to open surgery (0.55 [0.49, 0.62], *p* < 0.001).

**Table 4:** Multivariate logistic regression model for conversion to open among elective cholecystectomy

Characteristic	OR (95% CI)^1^	p-value
Modality		
Lap	–	
Robotic	0.48 (0.40, 0.57)	** < 0.001**
Gender		
F	–	
M	2.60 (2.32, 2.93)	** < 0.001**
Age group		
18–34 years	–	
35–44 years	1.81 (1.33, 2.48)	** < 0.001**
45–64 years	3.55 (2.75, 4.65)	** < 0.001**
65 + years	5.35 (3.95, 7.31)	** < 0.001**
Discharge year		
2020	–	
2021	1.00 (0.89, 1.13)	> 0.90
2022	0.84 (0.68, 1.03)	0.10
Indication for surgery		
Without obstruction	–	
Pancreatitis	0.62 (0.37, 0.96)	**0.044**
Sepsis	1.78 (1.31, 2.37)	** < 0.001**
With obstruction	2.83 (2.04, 3.83)	** < 0.001**
Overweight or obesity		
Not overweight or obesity	–	
Obesity	1.12 (0.98, 1.27)	0.086
Overweight	1.07 (0.91, 1.25)	0.40
Diabetes	1.21 (1.05, 1.39)	**0.007**
Payor type		
Commercial	–	
Medicaid	1.73 (1.42, 2.10)	** < 0.001**
Medicare	1.56 (1.28, 1.91)	** < 0.001**
Others	1.09 (0.85, 1.38)	0.50
Hospital type		
Academic/teaching	–	
Community hospital	0.37 (0.33, 0.42)	** < 0.001**
Others	0.16 (0.08, 0.28)	** < 0.001**
Region		
Northeast	–	
Midwest	1.04 (0.87, 1.26)	0.60
South	1.14 (0.96, 1.36)	0.13
West	0.95 (0.79, 1.14)	0.60
Surgeon volume		
Low volume (≤ 35/yr)	—	
High volume (> 35/yr)	0.55 (0.49, 0.62)	** < 0.001**
Missing	0.94 (0.29, 2.27)	> 0.90

Bolded p-values indicate statistical significance with *p* < 0.05

^1^*OR* odds ratio, *CI* confidence interval

A subgroup analysis of patients with or without sepsis and obstruction as the reason for cholecystectomy was conducted (Table 5). In patients with obstruction or sepsis, RCHOLE had reduced odds of conversion to open (0.37 [0.16, 0.84], *p* < 0.018). The same was true for patients without obstruction or sepsis (0.50 [0.41, 0.61],* p* < 0.001) although overall rates of conversion were higher in both groups for patients with obstruction or sepsis (LCHOLE 4.1% vs RCHOLE 1.6%) than without (LCHOLE 1.1% vs RCHOLE 0.6%).

**Table 5:** Conversion to open rates of subgroup analysis in patients with or without obstruction and sepsis after IPTW

	Obstruction/Sepsis					Without Obstruction/Sepsis		
Characteristic	Lap, N = 2,155^1^	Robotic, N = 405^1^	OR (95% CI)^2^	p-value	Lap, N = 90,961^1^	Robotic, N = 23,187^1^	OR (95% CI)^2^	p-value
Conversion to open				0.018				< 0.001
Not converted to open	2066 (95.9%)	399 (98.4%)	–		89,915 (98.9%)	23,051 (99.4%)	–	
Converted to open	89 (4.1%)	6 (1.6%)	0.37 (0.16, 0.84)		1,046 (1.1%)	135 (0.6%)	0.50 (0.41, 0.61)	

^1^n (%)

^2^*OR* odds ratio, *CI* Cconfidence interval

## Discussion

This study represents a large-scale, multi-institutional sampling of data for comparison of elective LCHOLE and RCHOLE. Patient data was fractionated into multiple covariates that were balanced using inverse probability treatment weighing between groups. In addition, multiple payor types, institution types, and regions were included.

Our results suggest a consistent benefit of RCHOLE over LCHOLE. In a balanced population of elective cholecystectomy patients, the data suggests an advantage of RCHOLE for decreased conversion rates, readmissions, hospital-acquired conditions, bile duct injuries, costs, and length of stay. However, the data also indicates that RCHOLE has a longer operative time. LCHOLE has a higher risk of converting to open surgery, and once open, also suggests an increased risk of performing a subtotal cholecystectomy rather than complete cholecystectomy. These results are slightly different from our findings in the acute gallbladder pathology population, in which we found a significant increase in the use of robotic subtotal cholecystectomy compared to laparoscopic [13]. We believe this difference is attributable to the decreased difficulty in the elective cases compared to the more urgent cases. In the elective setting, rates of subtotal cholecystectomy were approximately 0.2% in both groups, whereas in the acute setting, laparoscopic subtotal cholecystectomy was performed in 0.5% of cases with conversion to open in 1.9% of cases, while robotic subtotal cholecystectomy was performed in 0.7% of cases with conversion to open in 1.1% of cases [13]. The overall minimally invasive approach to the “difficult gallbladder” through various sub-total techniques has evolved with time, but is still not well delineated and we need additional studies to help pave the way forward [15–19].

The recent manuscript by Kalata and coauthors has generated a great deal of concern regarding potentially elevated rates of common bile duct injury with robotic cholecystectomy. We did not observe the same findings, with very low rates of bile duct injury and leak noted in both our current study (0.02% laparoscopic, 0.0% robotic) and our prior publication on the acute cholecystectomy comparison (0.1% laparoscopic, 0.0% robotic) [13]. Notably, when examining bile duct injury specifically, we observed zero injuries in the robotic group compared to 0.02% in the laparoscopic group (*p* = 0.009), and this difference persisted after adjustment. Kalata also notes an increased risk of bile duct injuries in elective cases, which our data also does not support [14]. In neither of our studies does the conversion rate compare to the apparent conversion rate in the Kalata study, which comprised 20% of the open cholecystectomies. We also studied the effect of surgeon volume in our data, noting a decreased rate of conversion to open with surgeons performing more than 35 cholecystectomies annually. Additional data regarding patient allocation for RCHOLE or LCHOLE was unable to be extrapolated from this data set.

While multiple studies have examined the clinical outcomes of robotic cholecystectomy, the cost of robotic chole remains an underexplored lingering concern. Our activity-based costing analysis demonstrates that RCHOLE was associated with cost savings when compared to LCHOLE, a finding which has not previously been published. While activity based costing analysis provides a breakdown of resource allocation and cost efficiency between modalities, it is important to note the cost of conversions to open surgery presents an additional financial burden of $23,358, or a 259% increase, not captured in the activity based costing framework [9]. Our comprehensive cost analysis combined with the observed 64% reduction in conversions for the RCHOLE group relative to the LCHOLE group reinforces RCHOLE’s cost-effectiveness.

This study demonstrates enhanced outcomes, including reduced readmission rates, conversion rates, bile duct injury rates, and cost. The robotic approach was not only associated with reduced conversion rates to open, but also decreased rates of subtotal cholecystectomy once converted. This suggests that the robotic approach’s improved HD 3D visualization and wristed articulation is advantageous in appraising the likelihood of a successful complete cholecystectomy instead of an incomplete, subtotal cholecystectomy when compared to laparoscopy.

The main strength of this study is that it is an analysis of a large sample of patients who underwent minimally invasive cholecystectomy across the US. There remain some notable limitations, however. As a retrospective study relying on the ICD and CPT codes extracted from hospital EHR, it is subject to coding insufficiency and inaccuracies; one example is the inability to classify the reason for conversion from laparoscopic or robotic to open surgery and the inability to use validated complication stratification schemes such as the Clavien-Dindo Classification or the Comprehensive Complication Index. Data validation and integrity checks performed by individual hospitals before data submission may mitigate the issue, though possible variation in coding behaviors across hospital systems still cannot be excluded. The study is also limited by potential selection bias in allocating patients to receive LCHOLE or RCHOLE. Performing IPTW analysis to balance different covariates reduces the impact of this bias, but unobserved confounding factors are still likely to exist. These include criteria for selection of their surgical approach and certain predictors for difficult cholecystectomy. Additionally, despite the study patients’ treatment in various types of hospitals across different geographic regions in the US, this study’s results may not be generalizable to the entire country as hospitals must individually opt into this database to understand national benchmarks, performance improvement and support quality initiatives.

## Conclusions

In our study we found that, in the elective setting, robotic cholecystectomy was associated with reduced rates of conversion to open surgery and reduced rates of open subtotal cholecystectomy when conversion was required. Robotic cholecystectomy was associated with reduced length of stay, bile duct injury rate, readmission rate, cost, and hospital acquired conditions when compared to laparoscopy. Thus this study demonstrates superiority of robotic cholecystectomy with regards to conversion rate, safety profile, and cost.

## Supplementary Information

Below is the link to the electronic supplementary material.

Supplementary file1 (DOCX 34 KB)

Supplementary file2 (XLSX 20 KB)

## Data Availability

The Intuitive Custom Hospital Analytics Database is proprietary and thus cannot be posted publicly.
